# Rosacea: Pathogenesis and Therapeutic Correlates

**DOI:** 10.1177/12034754241229365

**Published:** 2024-03-07

**Authors:** Ryan S. Q. Geng, Adrienn N. Bourkas, Asfandyar Mufti, R. Gary Sibbald

**Affiliations:** 1 Temerty School of Medicine, University of Toronto, Toronto, ON, Canada; 2 School of Medicine, Queen’s University, Kingston, ON, Canada; 3 Division of Dermatology, Department of Medicine, University of Toronto, Toronto, ON, Canada; 4 Dalla Lana School of Public Health and Division of Dermatology, Department of Medicine, University of Toronto, Toronto, ON, Canada

**Keywords:** rosacea, pathogenesis, treatment

## Abstract

Rosacea is a chronic inflammatory condition of which there is no cure. The pathogenesis of rosacea is likely multifactorial, involving genetic and environmental contributions. Current understanding suggests that pro-inflammatory pathways involving cathelicidins and inflammasome complexes are central to rosacea pathogenesis. Common rosacea triggers modulate these pathways in a complex manner, which may contribute to the varying severity and clinical presentations of rosacea. Established and emerging rosacea treatments may owe their efficacy to their ability to target different players in these pro-inflammatory pathways. Improving our molecular understanding of rosacea will guide the development of new therapies and the use of combination therapies.

## Introduction

Rosacea is a chronic inflammatory condition that has a high prevalence among fair-skinned adults of Northern European descent.^1^ Rosacea was originally classified by the National Rosacea Society Expert Committee according to a subtypes approach, with 4 predominant subtypes: erythrotelangiectatic, papulopustular, phymatous, and ocular. Erythrotelangiectatic rosacea is characterized by facial erythema, often with telangiectasias present. Papulopustular rosacea presents with facial erythema and a variable number of erythematous papules and pustules. Phymatous rosacea involves the thickening of the skin and hyperplasia of sebaceous glands, often in the nasal region (rhinophyma). Ocular rosacea often presents with blepharitis, conjunctivitis, and chalazion.^2^ Rosacea can also occur with other dermatoses.

However, rosacea is a complex condition where patients may not fit neatly into 1 subtype, can present with multiple subtypes, progress between subtypes, and occur with other dermatoses. Classification of diagnoses into rigid subtype categories may impede assessment of severity, prevent full coverage of clinical presentation, and be detrimental to patient outcomes. As such, a more patient-focused phenotypes approach was developed by the Global Rosacea Consensus panel, which is now the predominant approach used in clinical practice. In the phenotyping approach, the presence of phymatous changes or persistent erythema satisfy the conditions of a cutaneous rosacea diagnosis, with major features including papules/pustules, flushing/blushing, and telangiectasis, and minor features including stinging, burning, dryness, and edema. Ocular rosacea features include lid margin telangiectasia, blepharitis, keratitis, conjunctivitis, and anterior uveitis. The phenotyping approach also takes into account the severity of rosacea features.^3,4^

Most global population studies on rosacea report a prevalence of 1% to 3%. However, there is a wide range, with studies conducted in the Faroe Islands reporting a low 0.09% with Estonia reporting a higher 22%.^5^ Although fair-skinned individuals and women have the greatest risk for rosacea, it is important to recognize that rosacea also occurs in darker-skinned individuals.^6^ In a 1993 to 2010 US National Ambulatory Medical Care Survey analysis of the racial distribution of patients with rosacea, it was found that of all patients diagnosed with rosacea, 3.9% were Hispanic, 2.3% were Asian or Pacific Islander, and 2.0% were Black.^7^ It is important to note that the lower prevalence of rosacea in individuals with skin of color may be partly attributed to underdiagnosis due to the difficulty observing erythema and other rosacea features in darker skin.^8^

**Figure 1.**
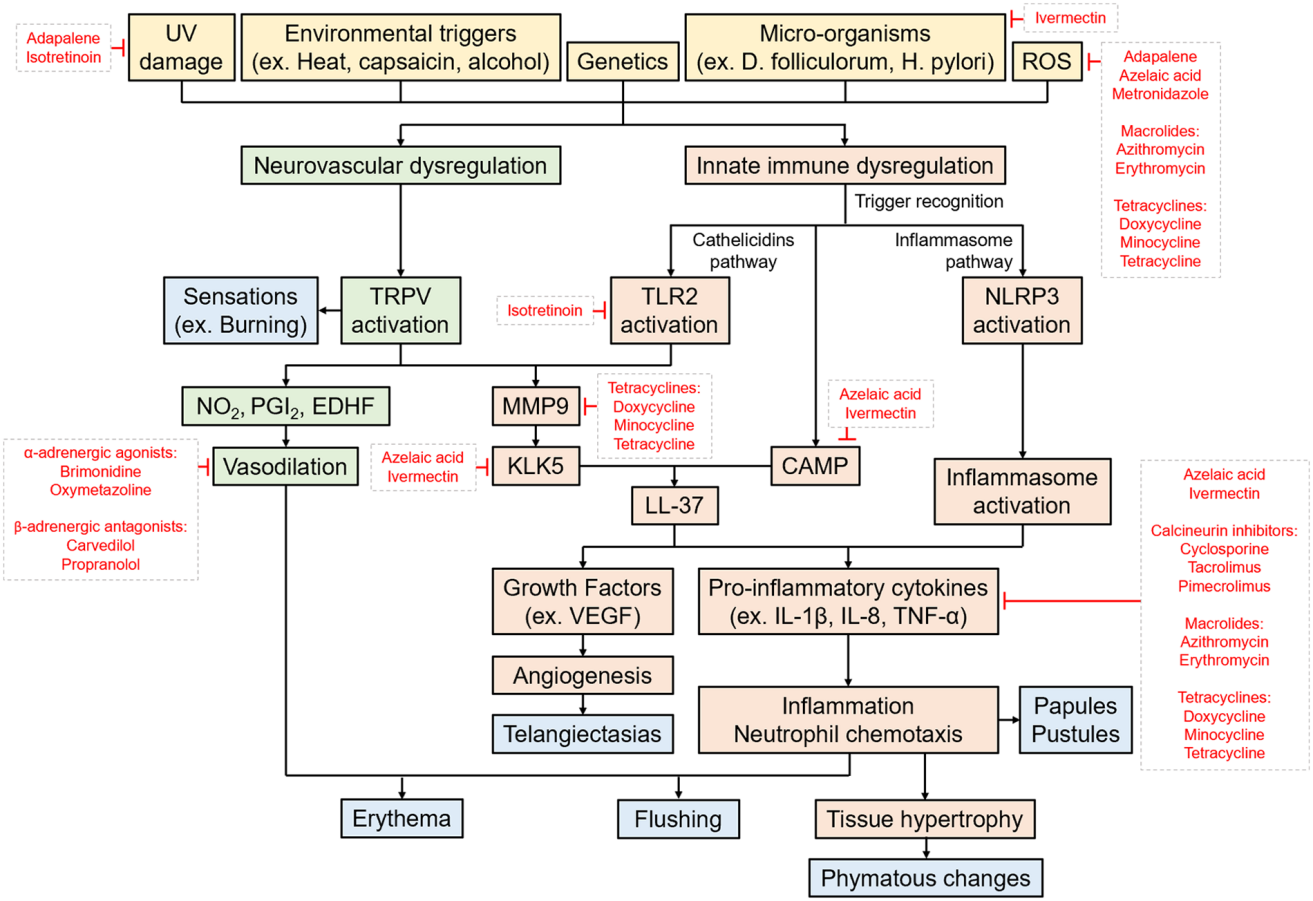
Schema of the risk factors, triggers, and pathogenesis pathways of rosacea. The pathogenesis of rosacea likely involves a complex interplay between several factors and pathways, including neurovascular dysregulation and innate immune system dysregulation involving cathelicidins and inflammasomes. These processes then give rise to the symptoms and clinical features of rosacea, depicted in blue. Known targets of medications currently used in treating rosacea are indicated.

While there is currently no cure for rosacea, several treatment options are available focusing on symptom management. With advances in research into the factors involved in the pathogenesis of rosacea, we are beginning to appreciate the complexities of rosacea at a molecular level as well. In this review, the inflammatory pathways and how established and emerging rosacea treatments interact with these pathways will be discussed. A schema of rosacea pathogenesis is provided in Figure 1.

## Receptors Involved in Rosacea Pathogenesis

The triggering factors of rosacea are well characterized and likely exert their pathogenic effects by activating receptors that contribute to and potentiate the inflammation observed in rosacea. Receptors that are upregulated in the skin of rosacea patients and their activation by common rosacea triggers are summarized in Supplemental Table S1.

## Neurovascular Dysregulation

Rosacea pathogenesis is likely to be multifaceted with neurovascular dysregulation being a major component. Key to the dysregulation of neurovascular processes are the transient receptor potential channels (TRPs), which are nonselective Ca^2+^ channels with sensory and signalling roles. TRPs are expressed in a variety of cell types including sensory neurons, mast cells, endothelial cells, and keratinocytes. TRPs respond to a wide range of stimuli, some of which are common triggers in rosacea. For example, transient receptor potential vanilloid (TRPV)-1 is responsive to heat, emotional stress, and capsaicin, among others. TRPV1 activation results in painful burning sensations.^9^ Activation of TRPs also leads to the activation of vasodilatory pathways, potentially resulting in the flushing and erythema observed in rosacea. For example, TRPV4 activation results in the production of nitric oxide (NO), prostaglandin I2, and endothelium-derived hyperpolarizing factor.^10^

## Innate Immune Dysregulation

### Cathelicidins Pathway

Cathelicidins is a family of antimicrobial peptides expressed in the skin in many vertebrates and have been implicated in rosacea pathogenesis.^11^

#### Toll-like receptor 2

Toll-like receptor (TLR)-2 is 1 of the 10 TLRs identified in humans. As a pattern recognition receptor (PRR), it recognizes molecular patterns found in microbial components to trigger immune responses against them. TLR2 expression is found in the plasma membranes of a variety of cells in the skin including keratinocytes, mast cells, and endothelial cells.^12^ TLR2 expression has been found to be elevated in the skin of rosacea patients.^13^ Activation of TLR2 leads to the production of cathelicidin (LL-37) and cytokines in the pro-inflammatory cathelicidins pathway are described in more detail in this article.

#### Pathway

Human cathelicidin antimicrobial peptide (CAMP) is intimately involved in the pathogenesis of rosacea, with both immunomodulatory and angiogenic properties. The expression of CAMP in keratinocytes is activated through either a vitamin D-dependent pathway involving the interaction of vitamin D with its receptor to promote CAMP expression, or a vitamin D-independent pathway where CAMP expression is induced by endoplasmic reticulum stress.^14^ Recently, mast cells have been implicated as a primary source of cathelicidins in rosacea.^15^

Rosacea patients exhibit abnormally high levels of CAMP and kallikrein-5 (KLK5), which is a serine protease that cleaves CAMP into bioactive fragments including LL-37.^16^ KLK5 is expressed in an inactive form that is activated on cleavage by matrix metalloproteases (MMP), including MMP9. Unsurprisingly, the skin of rosacea patients exhibits unusually high levels of MMP9.^17^ KLK5 activity is enhanced by TLR2 and TRPV4.^14,18^

The increased abundance of CAMP and KLK5 activity has been shown to increase the levels of bioactive cathelicidin fragments in rosacea patients. LL-37 levels were markedly increased and fragments not normally observed in healthy skin were also present.^19^ Increased levels of LL-37 promote reactive oxygen species (ROS) production by neutrophils, release of pro-inflammatory cytokines including interleukin (IL)-8, and angiogenic growth factors including vascular endothelial growth factor (VEGF) from keratinocytes by activating the epidermal growth factor receptor (EGFR) pathway. This could potentially explain the chronic inflammation and enhanced vascular growth observed in rosacea.^19-21^

### Inflammasome Pathway

Inflammasomes are multiprotein complexes of the innate immune system that trigger inflammatory responses through the activation of caspase-1.^22^

#### NLRP3 receptors

Nucleotide-binding oligomerization do-main, leucine rich repeat, and pyrin domain containing 3 (NLRP3) is a cytosolic PRR expressed in immune cells and keratinocytes that detects and triggers immune reaction in response to cellular damage.^23^ NLRP3 is involved in the activation of inflammasome complexes that result in the production of pro-inflammatory cytokines in the inflammasome pathway described in more detail in this article.

#### Pathway

Inflammasomes are activated by PRRs including NLRP3, which responds to particulate, bacterial, and protozoan matter. Inflammasome receptor activation results in the recruitment and cleavage of caspase-1 into its active form. Caspase-1 subsequently cleaves pro-inflammatory cytokines including IL-1β and IL-18 into their active forms.^22^ NLRP3, caspase-1, and IL-1β are known to be elevated in rosacea patients, implicating the inflammasome in rosacea pathogenesis.

It is important to note that the cathelicidins and inflammasome pathways interact with one another. TLR2 increases the expression of pro-IL-1β, while LL-37 enhances the processing of pro-IL-1β by the inflammasome.^23,24^ The enhanced activity of IL-1β results in upregulation of other pro-inflammatory cytokines including IL-8 and tumor necrosis factor (TNF)-α, angiogenesis, and chemotaxis of neutrophils.^23^ This provides an explanation for the enhanced vascularization and inflammation observed in the skin of rosacea patients. Recently, the use of a NLRP3-specific inhibitor reduced LL-37-triggered rosacea symptoms, demonstrating the role of NLRP3 in rosacea pathogenesis.^25^

## Adaptive Immune Dysregulation

While the bulk of rosacea pathogenesis centres around vascular hyperactivity and dysregulation of the innate immune system, evidence of adaptive immune involvement is emerging. In one study, rosacea skin biopsies exhibited elevated levels of CD4+ T-cells, particularly Th1 and Th17 subtypes. Interestingly, the clinical subtypes of rosacea exhibited differences in CD4+ elevation, with the papulopustular subtype displaying the greatest elevation.^26^

## Risk Factors and Triggers of Rosacea

**Figure 2.**
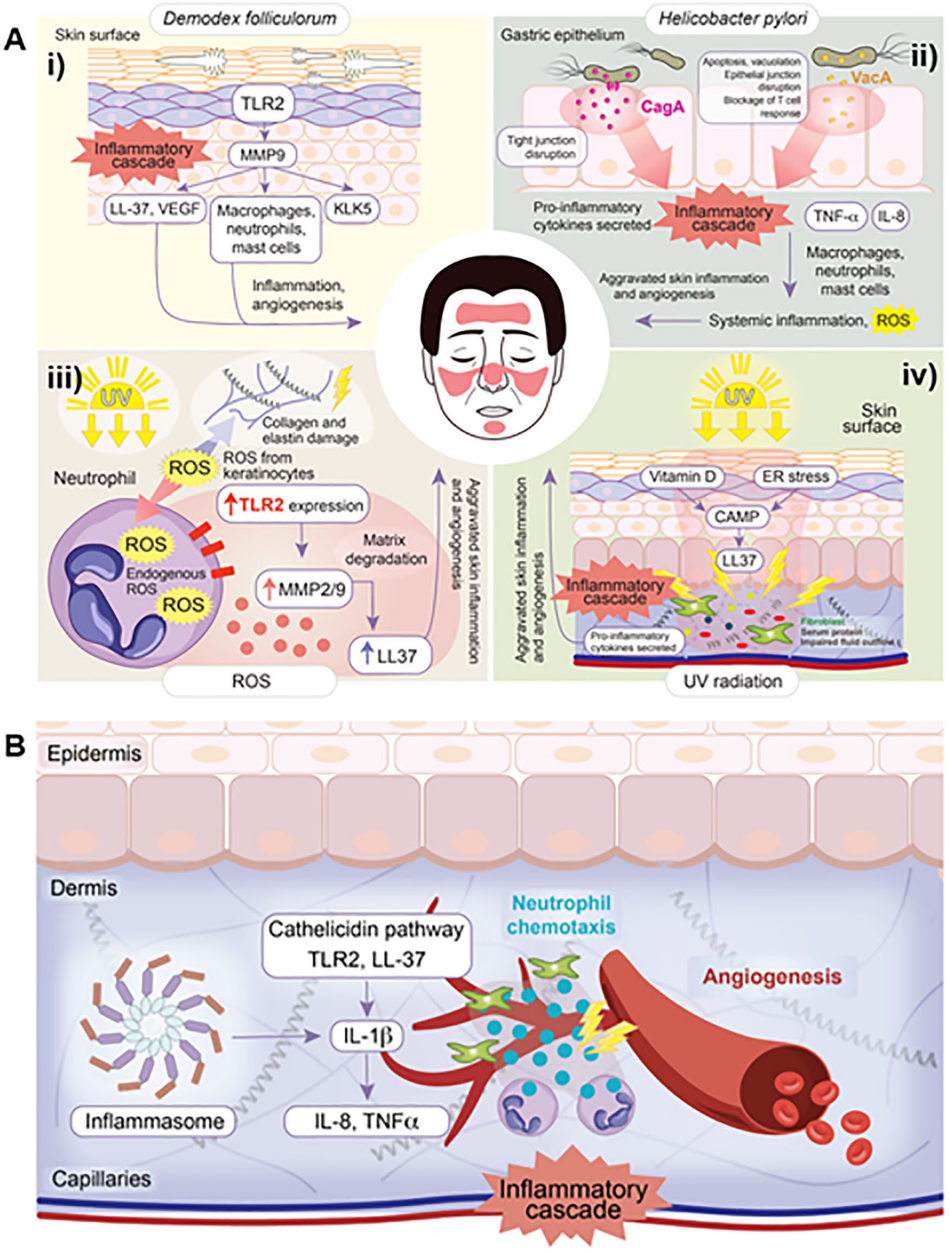
(A) Proposed cathelicidins pathway believed to be implicated in the pathogenesis of rosacea. Production of LL-37 leads to several physiological effects including angiogenesis, vasodilation, inflammation, and matrix degradation. (i) High densities of *Demodex folliculorum* upregulates the expression of TLR2 in sebocytes. Enhanced TLR2 activity leads to increased KLK5 activity and production of LL-37. (ii) CagA+ and VacA+ *Helicobacter pylori* strains stimulate mast cell activation and subsequent increase in histamines and prostaglandins, promoting inflammation. (iii) Through MMP2 upregulation and TLR2 signalling, ROS promote matrix degeneration and increase LL-37 production. (iv) UV promotes expression of CAMP in keratinocytes via vitamin D-dependent and ER stress-induced pathways. CAMP can then be cleaved into the bioactive LL-37 fragments. (B) Proposed inflammasome pathway believed to be implicated in the pathogenesis of rosacea. Inflammasome activation results in several physiological effects including chemotaxis of neutrophils, angiogenesis, and inflammation. The cathelicidins and inflammasome pathways are intricately linked. TLR2, toll-like receptor 2; KLK5, kallikrein-5; CagA+, cytotoxin-associated gene A positive; VacA+, vacuolating cytotoxin A positive; MMP2, matrix metalloprotease 2; ROS, reactive oxygen species; UV, ultraviolet; CAMP, cathelicidin antimicrobial peptide; ER, endoplasmic reticulum.

There are several risk factors and triggers of rosacea that facilitate rosacea pathogenesis through activating or modulating the neurovascular or innate immune processes described here. As previously discussed, common environmental triggers including heat, capsaicin, and alcohol exert their effects through activating TRPs. In this article, genetic predisposing factors and other triggers that modulate the innate immune processes will be discussed. A diagram summarizing the cathelicidins and inflammasome pathways and their interactions with rosacea triggers is provided in Figure 2.

### Genetics

The evidence supporting a genetic link in rosacea has been steadily building over the years. Population studies have shown that rosacea does not exhibit a uniform distribution across all populations but is instead more prevalent in fair-skinned individuals, particularly those of Northern European descent.^1^ Approximately 30% to 40% of rosacea patients also had a relative with rosacea.^1,27^ Twin studies have also found that there is a 46% genetic contribution to the development of rosacea.^28^

Perhaps the strongest piece of evidence comes from a genome-wide association study which identified 2 genes associated with rosacea. Both HLA-DRA (human leukocyte antigen class II histocompatibility antigen, DR alpha chain) and BTNL2 (butyrophilin-like 2, major histocompatibility complex class I associated) have previously been implicated in other inflammatory conditions such as multiple sclerosis and sarcoidosis.^29^

### Microorganisms

#### Demodex folliculorum

*D. folliculorum* mites are common inhabitants of human skin that live within pilosebaceous filaments. Humans are not born with these mites but are instead acquired through contact with other humans.^30^ The observation that *D. folliculorum* mites have a preference toward inhabiting the facial regions most commonly affected by rosacea and that an increase in mite density coincides with the clinical presentation of rosacea symptoms suggests a role for these mites in the pathogenesis of rosacea.^31^

Under normal conditions, these mites do not trigger an immune response. At low densities, *D. folliculorum* mites suppress TLR2 expression in sebocytes to evade the host immune defenses. However, high densities of *D. folliculorum* mites stimulate upregulation of TLR2 and induce secretion of inflammatory mediators by sebocytes.^30,32^ The chitin exoskeleton of the mites also increases TLR2 activity.^17^ In a cross-sectional prospective study comparing rosacea patients with sex- and age-matched controls, the skin of rosacea patients had over a 5-fold increase in *D. folliculorum* density and increased expression of pro-inflammatory cytokines such as IL-8 and TNF-α.^33^

*D. folliculorum* mites are hosts to *Bacillus oleronius* gram-negative bacteria, which are released when the mites die. *B. oleronius* has been shown to produce antigenic proteins that trigger immune responses in patients with inflammatory and flushing and erythematic rosacea.^34^ *D. folliculorum* and *B. oleronius* numbers can be effectively reduced with ivermectin and doxycycline, respectively, while also reducing inflammation as both medications boast anti-inflammatory properties.^35,36^ Although *B. oleronius* is susceptible to doxycycline, it is usually only taken in an anti-inflammatory dose (<100 mg/day) rather than an antimicrobial dosage.

While it does appear that *D. folliculorum* mites may play a role in modulating the inflammatory cascade of rosacea, direct evidence suggesting a causal linkage between the mites and rosacea is lacking. Complicating matters further, resolution of rosacea symptoms through treatment with either oral tetracycline or topical 3% sulphur did not affect *D. folliculorum* populations.^31^

#### Helicobacter pylori

*H. pylori* infection of the gastric epithelium is common in humans and has a prevalence rate of around 80% in developing countries and 20% to 50% in developed countries. Like with *D. folliculorum*, humans are not born with *H. pylori* but instead acquire them through oral ingestion, usually during early childhood.^37^

Continuous gastric inflammation induced by cytotoxin-associated gene A (CagA) or vacuolating cytotoxin A-positive *H. pylori* strains has been shown to increase the levels of vasoactive substances such as histamine and prostaglandins through the activation and degranulation of mast cells.^31,38^ Similar to *D. folliculorum*, there is also an increase in pro-inflammatory cytokines including IL-8.^39^ This may contribute to the chronic inflammation and vasodilation observed in rosacea.

In a study investigating the prevalence of *H. pylori* in patients with rosacea, participants were compared with age- and sex-matched controls. The prevalence of *H. pylori* was 88% in rosacea patients and 65% in controls. However, among the *H. pylori*-positive subjects, there were twice as many rosacea patients with CagA+ strains compared to controls. Interestingly, eradication of the *H. pylori* infection with a week-long therapy using omeprazole, clarithromycin, and metronidazole resulted in resolution of rosacea symptoms and normalization of the pro-inflammatory cytokines IL-8 and TNF-α.^40^ This was corroborated by another study which found that eradication of *H. pylori* with amoxicillin, metronidazole, and bismuth subcitrate resulted in significant improvements in rosacea symptoms.^41^ The use of metronidazole and oral antibiotics, common rosacea treatments, in both of these studies complicates the findings as it is unclear if the resolution of rosacea symptoms is due to eradication of *H. pylori* or the anti-inflammatory and antioxidative properties of the drugs themselves. Further complicating the potential involvement of *H. pylori* in rosacea pathogenesis is the results from 2 other independent studies that eradicated *H. pylori* but observed no improvement in rosacea symptoms.^31^

### UV Radiation

Histopathologic investigations have previously implicated the involvement of endothelial and dermal matrix damage in rosacea.^42^ Dermal matrix degradation, vasculature damage, and loss of perivascular structural support can result in an accumulation of serum proteins, pro-inflammatory mediators, and impair fluid outflow. All this would result in a persistence of the erythema, edema, and development of thread-like vessels observed in rosacea.^1,31,43^

The theories surrounding the involvement of endothelial and dermal matrix damage in the pathogenesis of rosacea revolve around UV light exposure. UV radiation is known to damage superficial cutaneous vasculature and the elastic and collagen fibres that comprise the dermal matrix.^31^ UV radiation also results in the upregulation of MMPs involved in collagenolysis, inflammation, and angiogenesis.^44^ In particular, MMP9 has been found to be upregulated in rosacea patients.^17^ MMP9 activity leads to the release of pro-inflammatory cytokines including IL-8, which inhibits the production of collagen and further activate MMP9, creating a chronic inflammatory cycle.^45^

### Reactive Oxygen Species

ROS are highly reactive chemical compounds that can directly damage biomolecules. Rosacea patients have been shown to exhibit higher levels of ROS in their skin compared to controls. ROS appears to contribute to rosacea pathogenesis by propagating the inflammatory cascade.^17^

ROS are formed through the interaction between UV radiation and atomic oxygen. When formed, ROS can activate TLR2 signalling and increase expression of MMP2, leading to enhanced production of LL-37 and matrix degradation.^20^ ROS itself can also cause oxidative damage to the basic building blocks of the extracellular matrix including collagen and elastin. As such, the role of ROS in rosacea pathogenesis highlights the importance of sun protection and explains the effectiveness of rosacea medications that have antioxidative properties.

## Targets of Rosacea Treatments

Rosacea is a chronic inflammatory condition of which there is currently no cure. However, there exists several treatment options focusing on symptoms reduction, most of which are pharmacological in nature. The molecular mechanisms of action of rosacea treatments will be discussed here. A summary of rosacea treatments and their targets is provided in Supplemental Table S2.

### Established Treatments

#### Adrenergic receptor agonists

Oxymetazoline and brimonidine are alpha-1 and alpha-2 adrenergic receptor agonists, respectively. When applied topically, both compounds relieve the facial erythema observed in rosacea by exerting a local vasoconstriction effect. This is achieved through binding to the adrenergic receptors of vascular smooth muscle, stimulating contraction.^46^ Unlike the other treatments discussed, oxymetazoline and brimonidine only provide temporary symptoms relief, effective for up to 12 hours.

#### Adrenergic receptor antagonists

Carvedilol and propranolol are nonselective beta-adrenergic receptor antagonists. Unlike alpha adrenergic receptors, beta receptor activation promotes vasodilation. By inhibiting beta-adrenergic receptors on vascular smooth muscle, vasoconstriction is achieved. Studies have shown propranolol to be effective in relieving facial flushing, while carvedilol has demonstrated effectiveness in reducing erythema.^47,48^ Carvedilol is also able to reduce ROS production and scavenge free ROS, which may further contribute to its effectiveness in treating rosacea.^49^

#### Azelaic acid

Azelaic acid is a naturally occurring dicarboxylic acid that has proven effectiveness in treating papules and pustules and erythema in rosacea. The efficacy of azelaic acid appears to involve modulating gene expression, where it was found to reduce CAMP and KLK5 mRNA in the skin of rosacea patients.^50^ Azelaic acid also has antioxidative properties, where it reduces ROS production by neutrophils. This is achieved through inhibiting nicotinamide adenine dinucleotide phosphate (NADPH) oxidase, which is the enzyme responsible for the production of most neutrophilic ROS. Azelaic acid also protects cells from oxidative damage through the scavenging of ROS.^51^ Azelaic acid has also been shown to reduce IL-1β and TNF-α expression and secretion by keratinocytes.^52^

#### Calcineurin inhibitors

Cyclosporine, pimecrolimus, and tacrolimus are calcineurin inhibitors effective in treating rosacea due to their immunosuppressive properties. Cyclosporine in the form of an ophthalmic emulsion is often used to treat ocular manifestations, while pimecrolimus and tacrolimus are available as a cream or ointment, respectively, for the treatment of cutaneous lesions. As an example, the efficacy of cyclosporine is due to reducing the production of pro-inflammatory cytokines by T-lymphocytes in the conjunctiva.^53,54^ However, cyclosporine has also demonstrated immunosuppressive properties against monocytes, where it reduced their production of IL-1β, IL-8, and TNF-α.^55^

#### Ivermectin

Ivermectin is a semisynthetic antiparasitic drug derived from avermectin that has demonstrated effectiveness in treating papules and pustules.^56^ The effectiveness of ivermectin in treating rosacea is believed to involve both its antiparasitic and anti-inflammatory properties. Ivermectin eliminates parasites including *D. folliculorum* by binding to γ-aminobutyric acid (GABA) receptors in motor synapses, causing paralysis.^36^ In a group of 20 patients treated with topical ivermectin 1.0% once-daily, the density of *D. folliculorum* mites were reduced by week 6.^57^ In an experimental study on human epidermal keratinocytes, ivermectin was found to reduce expression of CAMP and KLK5.^58^ Ivermectin has also been shown to decrease production of IL-1β and TNF-α.^59^

#### Macrolides

Macrolides are a class of bacteriostatic antibiotics with members including erythromycin, clarithromycin, and azithromycin. Macrolides have demonstrated effectiveness in alleviating both cutaneous and ocular manifestations of rosacea. The efficacy of macrolides involves both its anti-inflammatory and antioxidative properties. Macrolides have been shown to reduce production of IL-1β, IL-8, and TNF-α by neutrophils and reduce ROS levels in rosacea patient skin.^60^

#### Metronidazole

Metronidazole is a member of the nitroimidazole class of anaerobic antibiotics that exhibits both antibiotic and antiprotozoal properties. Metronidazole is effective in treating papules and pustules, and also has limited effectiveness in treating erythema.^61^ While originally administered orally for treatment of anaerobic bacterial infections around the body, the efficacy of topical metronidazole in rosacea does not appear because of its antibacterial effects. In a group of 20 patients treated with metronidazole 1.0% for 1 month, there was no meaningful decrease in the number of bacteria harboured in the skin even after rosacea symptoms improved.^62^ Instead, metronidazole appears to be beneficial in managing rosacea through the inhibition of ROS generation by neutrophils and scavenging existing ROS.^63^ Furthermore, metronidazole appears to have anti-inflammatory properties through the inhibition of T-cells.^64^

#### Retinoids

Retinoids are a class of compounds derived from vitamin A commonly used for regulating skin turnover. Topical retinoids, such as the third-generation adapalene, have demonstrated effectiveness in treating papules and pustules.^65^ The first-generation isotretinoin when taken orally has shown efficacy in treating severe recalcitrant rosacea.^66^ The mechanism of action of retinoids in treating rosacea has not been well elucidated. However, studies have shown that adapalene is able to reduce ROS production by neutrophils, while isotretinoin reduced TLR2 expression in monocytes.^46,67^ Furthermore, retinoids promote connective tissue remodelling, repairing the contributions of UV radiation to rosacea pathogenesis.^46^

#### Tetracyclines

Tetracyclines are a class of antibiotic that exhibit bacteriostatic activity. Of the tetracycline antibiotics, the first-generation tetracycline and the second-generation doxycycline are commonly prescribed for treating rosacea. These tetracyclines are typically prescribed at a sub-antimicrobial dose, relying on its anti-inflammatory and antioxidative properties to alleviate rosacea symptoms. In one experimental study, doxycycline was shown to directly inhibit MMP activity and expression, suppressing KLK5 activation and subsequent LL-37 production.^68^ Doxycycline also reduces levels of IL-1β, IL-8, and TNF-α.^69^ In addition, doxycycline has demonstrated effectiveness in scavenging neutrophil derived ROS.^70^

### Treatments Under Investigation in Past 5 Years (2018)

Several treatments have been recently investigated for their efficacy in rosacea. These include newly developed agents and agents being used in treating other diseases but with emerging indications in rosacea. A table summarizing these newer treatments is provided (Supplemental Table S3) and further discussed here, with trials data provided where available.

#### Proteasome inhibitor (ACU-D1)

ACU-D1 is a 26S proteasome inhibitor assessed for efficacy in patients with moderate-severe rosacea. In the phase II trial, of the patients who applied ACU-D1 ointment twice daily for 14 weeks, 92% had reduced inflammatory lesions and 27% had 2+ grade Investigator’s Global Assessment (IGA) increase of clear and near-clear responses. Its efficacy is believed to be through the inhibition of the nuclear factor kappa-light-chain-enhancer of activated B cells pathway, reducing pro-inflammatory cytokines including IL-1 β, IL-6, and IL-8.^71,72^

#### B244

B244 is a spray containing a strain of *Nitrosomonas eutropha*, an ammonia oxidizing bacteria that produces NO. In the phase II trial, 67% of patients who applied 4 sprays twice-daily for 8 weeks demonstrated at least 1+ improvement in their Clinical Erythema Assessment score. The efficacy of *N eutropha* in reducing erythema in rosacea is likely due to its ability to downregulate pro-inflammatory cytokines, as NO is a vasodilator.^73,74^

#### CGB-400

CGB-400 is an ionic liquid/deep eutectic solvent composed of choline and geranic acid (CAGE) in a 1:2 ratio. In a phase I trial, patients who applied CAGE gel twice-daily for 12 weeks showed a 72% reduction in inflammatory lesions from baseline (*P* < .0001). The efficacy of CAGE is believed to be due to its ability to inhibit KLK5.^75,76^

#### Omiganan (CLS001)

Omiganan is an antimicrobial peptide, analogous to indolicidin. In the most recently completed phase III trial, patients who applied omiganan gel once-daily for 12 weeks observed a mean reduction of 18.1 inflammatory lesions compared to 13.5 for vehicle (*P* < .05). The mechanism of action of omiganan in rosacea is unclear but is believed to be anti-inflammatory due to the observation that omiganan reduced inflammatory lesion counts in acne trials.^77^

#### DMT310

DMT310 is a powder derived from the *Spongilla lacustris* freshwater sponge. Its efficacy in treating rosacea is attributed to its ability to downregulate pro-inflammatory cytokines IL-17A and IL-17F. A phase II trial assessing the efficacy of DMT310 mixed with hydrogen peroxide in reducing inflammatory lesions in rosacea is currently underway.^78^

#### Asivatrep (PAC-14028)

Asivatrep is a novel TRPV1 antagonist, making it potentially useful in the treatment of rosacea. The most recent phase II trial investigated the efficacy of asivatrep cream in reducing erythema and inflammatory lesions in rosacea, but results have not been released since the completion of the trial in 2016.^79^

#### Rifaximin

Rifaximin is a broad-spectrum antibiotic that is poorly absorbed orally and is thus used to act locally in the gut. A phase II trial investigating the efficacy of rifaximin delayed-release in reducing inflammatory lesions in rosacea was completed in 2022, but results have not been released.^80^ Given that rifaximin acts locally in the gut, its proposed efficacy in rosacea is likely related to the association between rosacea and small intestinal bacterial overgrowth (SIBO). In a separate trial, of the rosacea patients with SIBO treated with rifaximin, 46% reported cleared or markedly improved rosacea.^81^ Thus, rifaximin may be useful in treating rosacea patients with a background of SIBO.

#### Encapsulated benzoyl peroxide (S5G4T-1)

Benzoyl peroxide has long been used in the treatment of acne vulgaris, but concerns relating to skin irritation has prevented its use in rosacea. Benzoyl peroxide in an encapsulated form mitigates skin irritation and has been investigated for use in rosacea. In 2 identical phase III trials, patients who applied encapsulated benzoyl peroxide 5% cream for 12 weeks noted a 50.1% and 43.5% decrease in inflammatory lesion counts compared to 25.9% and 16.1% for vehicle, respectively. The precise mechanism of action of benzoyl peroxide in rosacea is unclear.^82^ Encapsulated benzoyl peroxide 5% cream was recently approved by the Food and Drug Administration (FDA) for treating inflammatory lesions in rosacea.

#### Sarecycline

Sarecycline is a tetracycline-class antibiotic that was recently approved for treating acne vulgaris. In a phase IV trial, patients treated with weight-adjusted dose of sarecycline once-daily for 12 weeks reported 80% reduction in inflammatory lesions compared to 60% for placebo. Similar to the mechanism of other tetracyclines, the efficacy of sarecycline in rosacea may be attributed to its anti-inflammatory properties.^83^

#### Secukinumab

Secukinumab is a monoclonal antibody targeting IL-17 being used in treating psoriasis. In a phase I/II trial, patients treated with 7 doses of subcutaneous secukinumab 300 mg over 16 weeks noted a median reduction of 5 inflammatory lesions (*P* = .01). However, it is unclear if this improvement is clinically relevant.^84^

#### Timolol

Timolol is a nonselective beta-adrenergic receptor antagonist that is typically used to treat glaucoma and infantile hemangiomas. Timolol may be beneficial in rosacea for its vasoconstrictive properties and has also been shown to reduce release of VEGF, MMP2, and MMP9.^85^ In a phase I trial, patients who applied timolol 0.5% gel twice-daily observed significant reduction in erythema (*P* < .001) by week 12, but with recovery of erythema at week 16.^86,87^

#### Lotilaner (TP-04)

Lotilaner is an antiparasitic that has been used in treated *Demodex* blepharitis and is being evaluated in rosacea due to its efficacy in eradicating *Demodex* mites. A phase II trial evaluating the efficacy of Lotilaner 2% gel in reducing erythema and inflammatory lesion counts is currently underway.^88^

#### Trametinib

Trametinib is a mitogen-activated extracellular signal-regulated kinase (MEK) inhibitor used as an anticancer therapy in melanoma. As a MEK inhibitor, trametinib has antiangiogenic properties that may be useful in treating erythema in rosacea. A phase I trial assessing the efficacy of trametinib cream in reducing erythema and edema in rosacea was completed in 2022, but results have not been posted.^89^

#### Minocycline

##### Minocycline gel (BPX-04, HY01)

Minocycline is a tetracycline-class antibiotic being investigated in rosacea. In a phase II trial, patients who applied minocycline 1% or 3% gel (HY01) once-daily for 16 weeks reported a mean reduction of 11.3 and 12.8 inflammatory lesions compared to 8 for vehicle (*P* < .05). The efficacy of minocycline is attributed to its ability to inhibit MMPs, reduce oxidative stress, and inhibit synthesis of NO.^90,91^

##### Minocycline foam (FMX103)

Minocycline has also been investigated in the form of a foam. In 2 identical phase 3 trials, patients who applied minocycline foam 1.5% once-daily for 12 weeks reported a mean reduction of 17.57 and 18.54 inflammatory lesions compared to 15.65 and 14.88 for vehicle, respectively (*P* < .01).^92-94^ Minocycline 1.5% foam has recently been approved for treating inflammatory lesions in rosacea in 2020.

##### Minocycline modified-release capsule (DFD-29)

Minocycline has also been assessed in rosacea systemically as an extended-release capsule and has demonstrated superiority over doxycycline. In 2 recently completed phase III trials, patients treated with minocycline 40 mg modified-release capsules once-daily for 16 weeks reported a mean reduction of 21.3 and 18.4 inflammatory lesions compared to 15.9 and 14.9 for doxycycline 40 mg and 12.2 and 11.1 for placebo (*P* < .001), respectively. Plans to seek FDA approval have been announced for the second half of 2023.^95,96^

#### Phosphodiesterase-4 (PDE4) inhibitors

##### PF-07038124

PF-07038124 is another PDE4 inhibitor mainly being investigated for efficacy in atopic dermatitis and plaque psoriasis. However, a phase II trial is currently in the recruiting phase to investigate the efficacy of PF-07038124 0.02% ointment in reducing inflammatory lesion count in rosacea.^97^

##### Roflumilast

Roflumilast is currently used in the treatment of chronic obstructive lung disease but being investigated for efficacy in rosacea due to its anti-inflammatory properties. A phase II trial investigating the effect of roflumilast cream on inflammatory lesions in rosacea was recently completed in 2023, with results yet to be posted.^98^

## Combination Therapies

Rosacea pathogenesis is complex and involves many different contributing factors. As discussed above, therapies effective in treating rosacea target different players in the pathogenesis pathways. This raises the exciting possibility of using combinations of synergistic therapies to simultaneously target multiple players in the pathogenesis pathway. Several studies have demonstrated the effectiveness of combinatorial therapies in rosacea.

In one study, oral doxycycline 40 mg was combined with metronidazole 1.0% gel and compared with metronidazole 1.0% gel alone. The combination group exhibited greater reductions in inflammatory lesion counts and erythema.^99^ In another study, oral doxycycline 40 mg was combined with ivermectin 1.0% cream and compared with ivermectin 1.0% cream alone. The combination treatment was more effective in reducing inflammatory lesions and erythema, and also had a faster onset of action.^100^

## Conclusion

Rosacea is a chronic inflammatory condition that likely involves vascular hyperactivity, cathelicidins, and inflammasome complex activation. While these pro-inflammatory pathways provide an explanation for rosacea pathogenesis, it is important to note that rosacea pathogenesis is likely not uniform across patients. Differences in genetics and environmental factors may alter the pathogenesis process and contribute to the differences observed in clinical presentation and severity.

Currently, novel therapies are being developed that target known factors involved in rosacea pathogenesis and therapies used in treating other conditions are being assessed for potential benefits in rosacea. By furthering our understanding of rosacea pathogenesis at the molecular level, we will be better equipped to develop safe and effective therapies to alleviate the burden of disease.

## Supplemental Material

sj-docx-1-cms-10.1177_12034754241229365 – Supplemental material for Rosacea: Pathogenesis and Therapeutic Correlates Supplemental material, sj-docx-1-cms-10.1177_12034754241229365 for Rosacea: Pathogenesis and Therapeutic Correlates by Ryan S. Q. Geng, Adrienn N. Bourkas, Asfandyar Mufti and R. Gary Sibbald in Journal of Cutaneous Medicine and Surgery
