# Supplementary material for: Rosacea: Pathogenesis and Therapeutic Correlates
Source: J Cutan Med Surg. 2024 Mar 7;28(2):178–89. doi: 10.1177/12034754241229365 (PMC11015710; doi:10.1177/12034754241229365)
Supplement: sj-docx-1-cms-10.1177_12034754241229365 – Supplemental material for Rosacea: Pathogenesis and Therapeutic Correlates [file sj-docx-1-cms-10.1177_12034754241229365.docx]

**Rosacea: Pathogenesis and therapeutic correlates**

**Supplemental Tables**

**Table S1.** Upregulated receptors in rosacea and their triggers. Adapted from Buddenkotte and Steinhoff, 2018.^1^

| **Receptor** | **Qualitative Triggers** |
| --- | --- |
| NLRP3 | Alcohol, emotional stress, exercise, micro-organisms, sun, wind |
| TLR2 | Alcohol, emotional stress, exercise, micro-organisms, sun |
| TRPA1 | Cold, formaldehyde |
| TRPV1 | Alcohol, capsaicin, emotional stress, exercise, heat |
| TRPV2 | Heat |
| TRPV3 | Heat |
| TRPV4 | Humidity, sun |

**Table S2.** Rosacea treatments and the targets they downregulate, listed in alphabetical order.

| **Treatment** | **Target(s)** |
| --- | --- |
| Adapalene | ROS, UV damage |
| Azelaic acid | CAMP, IL-1β, KLK5, ROS, TNF-α |
| Brimonidine, Oxymetazoline | Vasodilation |
| Carvedilol, Propranolol | ROS, Vasodilation |
| Cyclosporine | IL-1β, IL-8, TNF-α |
| Isotretinoin | TLR2, UV damage |
| Ivermectin | CAMP, D. folliculorum, IL-1β, KLK5, TNF-α |
| Macrolides | IL-1β, IL-8, ROS, TNF-α |
| Metronidazole | ROS |
| Tetracyclines | IL-1β, IL-8, MMPs, ROS, TNF-α |

**Table S3.** Pharmaceutical agents being assessed for efficacy in rosacea registered with Clinicaltrials.gov that have ongoing or completed trials within the past 5 years (2018 or later).

| **Agent** | **Sponsor** | **N patients** | **Phase** | **Status** |
| --- | --- | --- | --- | --- |
| ACU-D1 ointment | Accuitis, Inc. | 40 | Phase II^2^ | Completed |
| B244 spray | AOBiome LLC | 140 | Phase II^3^ | Completed |
| CGB-400 gel | CAGE Bio Inc. | 78 | Phase II^4^ | Completed |
|  |  | 27 | Phase I^5^ | Completed |
|  |  | 25 | Phase I^6^ | Completed |
| CLS001 gel | Maruho Co., Ltd. | 463 | Phase III^7^ | Completed |
|  |  | 307 | Phase III^8^ | Completed |
|  |  | 263 | Phase III^9^ | Completed |
|  |  | 240 | Phase II^10^ | Completed |
|  |  | 25 | Phase I^11^ | Completed |
| DMT310 powder | Dermata Therapeutics | 180 | Phase II^12^ | Active, not recruiting |
| PAC-14028 cream | Amorepacific Corp. | 216 | Phase II^13^ | Completed |
|  |  | 80 | Phase II^14^ | Completed |
| Rifaximin  delayed-release | Alfasigma S.p.A. | 216 | Phase II^15^ | Completed |
| *S5G4T-1 cream | Sol-Gel Technologies, Ltd. | 372 | Phase III^16^ | Completed |
|  |  | 361 | Phase III^17^ | Completed |
|  |  | 31 | Phase I^18^ | Completed |
| Sarecycline | Derm Research, PLLC | 100 | Phase IV^19^ | Completed |
| Secukinumab | Stanford University | 24 | Phase I/II^20^ | Completed |
| Timolol | Johns Hopkins University | 8 | Phase I^21^ | Completed |
| TP-04 gel | Tarsus Pharmaceuticals, Inc. | 30 | Phase II^22^ | Active, not recruiting |
| Trametinib | Albany Research Institute, Inc. | 12 | Phase I^23^ | Completed |
| **Minocycline** | | | | |
| BPX-04 gel | BioPharmX, Inc. | 30 | Phase I/II^24^ | Completed |
| *DFD-29 capsule | Journey Medical Corporation | 323 | Phase III^25^ | Completed |
|  |  | 330 | Phase III^26^ | Completed |
|  |  | 205 | Phase II^27^ | Completed |
|  |  | 60 | Phase I^28^ | Completed |
| *FMX103 foam | Vyne Therapeutics, Inc. | 504 | Phase III^29^ | Completed |
|  |  | 771 | Phase III^30^ | Completed |
|  |  | 751 | Phase III^31^ | Completed |
|  |  | 233 | Phase II^32^ | Completed |
| HY01 gel | Hovione Scientia, Ltd. | 270 | Phase II^33^ | Completed |
| **Phosphodiesterase-4 inhibitor** | | | | |
| PF-07038124 ointment | Icahn School of Medicine at Mount Sinai | 66 | Phase II^34^ | Recruiting |
| Roflumilast cream | Dermatology Consulting Services, PLLC | 40 | Phase II^35^ | Completed |

*Treatments approved by FDA in treating rosacea or with plans to seek FDA approval.

**References**

1. Buddenkotte J, Steinhoff M. Recent advances in understanding and managing rosacea. *F1000Res*. 2018;7doi:10.12688/f1000research.16537.1

2. Accuitis I. Safety and Efficacy of ACU-D1 in the Treatment of Acne Rosacea. 2018.

3. LLC AO, bioRasi LLC. A Study to Determine Safety and Efficacy of B244 in Subjects With Mild to Moderate Rosacea. 2019.

4. Inc CB, ethica Clinical Research I. CGB-400 Topical Gel for the Treatment of Inflammatory Lesions of Rosacea. 2021.

5. Inc CB, ethica Clinical Research I. CGB-400 for the Reduction of Facial Redness and Bumps and Blemishes. 2019.

6. Inc CB, ethica Clinical Research I. CGB-400 for the Reduction of Facial Redness. 2019.

7. Maruho Co L. Study to Evaluate the Safety and Efficacy of a Once-Daily CLS001 Topical Gel Versus Vehicle. 2017.

8. Maruho Co L. Study to Evaluate the Long-term Safety of a Once-Daily Omiganan Topical Gel. 2017.

9. Maruho Co L. Study to Evaluate the Safety and Efficacy of a Once-Daily CLS001 Topical Gel Versus Vehicle. 2017.

10. Maruho Co L. A Twelve Week Safety and Efficacy Study in Rosacea. 2014.

11. Maruho Co L. A 21 Day Pharmacokinetics Study in Papulopustular Rosacea. 2014.

12. Dermata T. DMT310-005 Topical in the Treatment of Acne Rosacea. 2022.

13. Amorepacific C. A Study to Evaluate the Safety and Efficacy of PAC-14028 Cream in Rosacea. 2016.

14. Amorepacific C. Study to Evaluate the Efficacy and Safety of PAC-14028 Cream in Rosacea Patients. 2013.

15. Alfasigma SpA, bioRasi LLC. Safety, Efficacy and Pharmacokinetics of Rifaximin in Patients With Moderate-to-severe Papulopustular Rosacea. 2022.

16. Sol-Gel Technologies L. A Study of S5G4T-1 in the Treatment of Papularpustular Rosacea. 2019.

17. Sol-Gel Technologies L. A Study of S5G4T-1 in the Treatment of Papulopustular Rosacea. 2019.

18. Sol-Gel Technologies L. Evaluation of Topical Encapsulated Benzoyl Peroxide on the Skin Microbiome and Skin Biophysical Properties. 2021.

19. Derm Research P. A Pilot Study on the Use of Seysara for Rosacea. 2020.

20. Stanford U. Open Label Study to Assess the Effect of Secukinumab in Moderate to Severe Papulopustular Rosacea. 2019.

21. Johns Hopkins U. Timolol for the Treatment of Acne and Rosacea. 2021.

22. Tarsus Pharmaceuticals I. Study of TP-04 in Participants With Papulopustular Rosacea. 2023.

23. Albany Research Institute I. Topical Mitogenic-Activated Protein Kinase (MAPK) Inhibition in Rosacea. 2022.

24. BioPharmX I. Open Label Phase 2 Feasibility Study of BPX-04 Topical Minocycline Gel in Rosacea. 2018.

25. Journey Medical C, Dr. Reddy's Laboratories L. A Randomized, Double-Blind Study to Assess the Safety, Efficacy and Tolerability of Oral DFD-29 Capsules for the Treatment of Rosacea. 2023.

26. Journey Medical C, Dr. Reddy's Laboratories L. A Study to Assess the Safety, Efficacy and Tolerability of Oral DFD-29 Capsules for the Treatment of Rosacea (MVOR-2). 2023.

27. Dr. Reddy's Laboratories L. A Controlled Study to Assess the Efficacy, Safety and Tolerability of Oral DFD-29 Extended Release Capsules. 2018.

28. Journey Medical C, Dr. Reddy's Laboratories L. Impact of DFD-29 on Microbial Flora of Healthy, Adult Human Subjects When Administered Over 16 Weeks. 2023.

29. Vyne Therapeutics I. A Study to Evaluate the Long-Term Safety of Topical Administration of FMX103 in the Treatment of Moderate to Severe Papulopustular Rosacea. 2019.

30. Vyne Therapeutics I, Premier Research Group p. A Study (Study 1) to Evaluate the Safety and Efficacy of FMX103 1.5% Topical Minocycline Foam in the Treatment of Facial Papulopustular Rosacea. 2018.

31. Vyne Therapeutics I, Premier Research Group p. A Study (Study 2) to Evaluate the Safety and Efficacy of FMX103 1.5% Topical Minocycline Foam in the Treatment of Facial Papulopustular Rosacea. 2018.

32. Vyne Therapeutics I. Safety and Efficacy Study of a Topical Minocycline Foam in Patients With Papulopustular Rosacea. 2016.

33. Hovione Scientia L. Study to Evaluate the Safety and Efficacy of Topical Minocycline Gel in Patients With Papulopustular Rosacea. 2019.

34. Icahn School of Medicine at Mount S, Pfizer. PDE4 Inhibition in Seborrheic Dermatitis and Papulopustular Rosacea. 2024.

35. Dermatology Consulting Services P. Evaluation of the Safety and Efficacy of Topical Roflumilast Cream in the Treatment of Facial Papulopustular Rosacea. 2023.
